# Multi-Level Kinetic Model Explaining Diverse Roles of Isozymes in Prokaryotes

**DOI:** 10.1371/journal.pone.0105292

**Published:** 2014-08-15

**Authors:** Jiri Jablonsky, Doreen Schwarz, Martin Hagemann

**Affiliations:** 1 Laboratory of Experimental Complex Systems, FFPW, University of South Bohemia, České Budějovice, Czech Republic; 2 Department of Plant Physiology, University of Rostock, Rostock, Germany; University of Delhi, India

## Abstract

Current standard methods for kinetic and genomic modeling cannot provide deep insight into metabolic regulation. Here, we developed and evaluated a multi-scale kinetic modeling approach applicable to any prokaryote. Specifically, we highlight the primary metabolism of the cyanobacterium *Synechococcus elongatus* PCC 7942. The model bridges metabolic data sets from cells grown at different CO_2_ conditions by integrating transcriptomic data and isozymes. Identification of the regulatory roles of isozymes allowed the calculation and explanation of the absolute metabolic concentration of 3-phosphoglycerate. To demonstrate that this method can characterize any isozyme, we determined the function of two glycolytic glyceraldehyde-3-phosphate dehydrogenases: one co-regulates high concentrations of the 3-phosphoglycerate, the other shifts the bifurcation point in hexose regulation, and both improve biomass production. Moreover, the regulatory roles of multiple phosphoglycolate phosphatases were defined for varying (non-steady) CO_2_ conditions, suggesting their protective role against toxic photorespiratory intermediates.

## Introduction

Cyanobacteria are a monophyletic group of oxygenic phototrophs in the phylum *Bacteria*. Recently, these prokaryotes have received increasing attention in applied research. Cyanobacterial production of bioenergy, such as hydrogen [1] or butanol [2], has been shown, and large-scale projects are believed to become part of sustainable and environmentally friendly bio-production in the future [3]. It is the computational biology which plays a key role in planning the cyanobacteria-based bio-production as well as in data evaluation and the understanding of cellular processes in cyanobacteria. However, some processes such dynamics of metabolic regulation, have been analyzed in-depth studies and methods analyzing the dynamics of metabolic regulation are missing. This deficiency may have been caused by a desire for analyzing the whole metabolic network, which was, and still is, a limiting factor for kinetic modeling. Consequently, genome-scale modeling has become a standard method for analyzing and filling the gaps in our knowledge of cyanobacteria metabolism and prokaryotes in general.

Most genome-scale models have used the model cyanobacterium *Synechocystis* sp. PCC 6803 [4], [5]. These models are useful in understanding cyanobacterial metabolism, especially the functional consequences of unknown pathways, “what if” analyses, day-night transition, and comparisons of multiple scenarios [6], [7]. However, genome-scale modeling has limitations. It cannot address the dynamics of metabolic regulation and the role of isozymes. It also suffers greatly from errors in the gene annotation and contains gaps that require additional input obtained from other organisms. Finally, genome-scale models are designed for maximal efficiency, whereas real systems are designed for sustained propagation in changing environments, which may lead to suboptimal metabolism. To overcome these problems, alternative approaches are needed.

Our preliminary study showed that a combination of genome-scale and kinetic models improves the model accuracy by implementing minor sink reactions for adjacent pathways [8]. However, the problem of unconstrained parameter estimation remains. A possible method to constrain the model is integrating transcriptomic and possibly other “omics” data. Since this approach does not work well for genome-scale modeling [9], the remaining option was to use multi-scale kinetic modeling. A pilot analysis of multi-scale kinetic modeling suggested that the key elements in the metabolic regulation of prokaryotes are isozymes. This analysis [10] explained the specific roles and possible evolution of phosphoglycerate mutases (PGMs). However, PGMs have a cardinal position in metabolism, and it is unknown whether this method is applicable for other, non-prominent isozymes.

This work aimed to present a multi-scale kinetic model of primary carbon metabolism for the model cyanobacterium *Synechococcus elongatus* PCC 7942 (hereafter referred to as *Synechococcus* 7942) and show its capabilities for explaining the metabolic and redox regulation of primary carbon metabolism. The model is constrained by metabolic and transcriptomic data, energy and redox levels (ATP/ADP, NADPH/NADP^+^, and NADPH/ATP ratios), CO_2_ level, and growth rate. First, the model was evaluated regarding whether it robustly mimics the experimental data. We then focused on the following questions: 1) Can we reliably predict cellular metabolic concentrations; 2) What is the role of isozymes and their position in the network; and 3) How is photorespiration integrated into the primary carbon metabolism system?

## Materials and Methods

**Figure 1:**
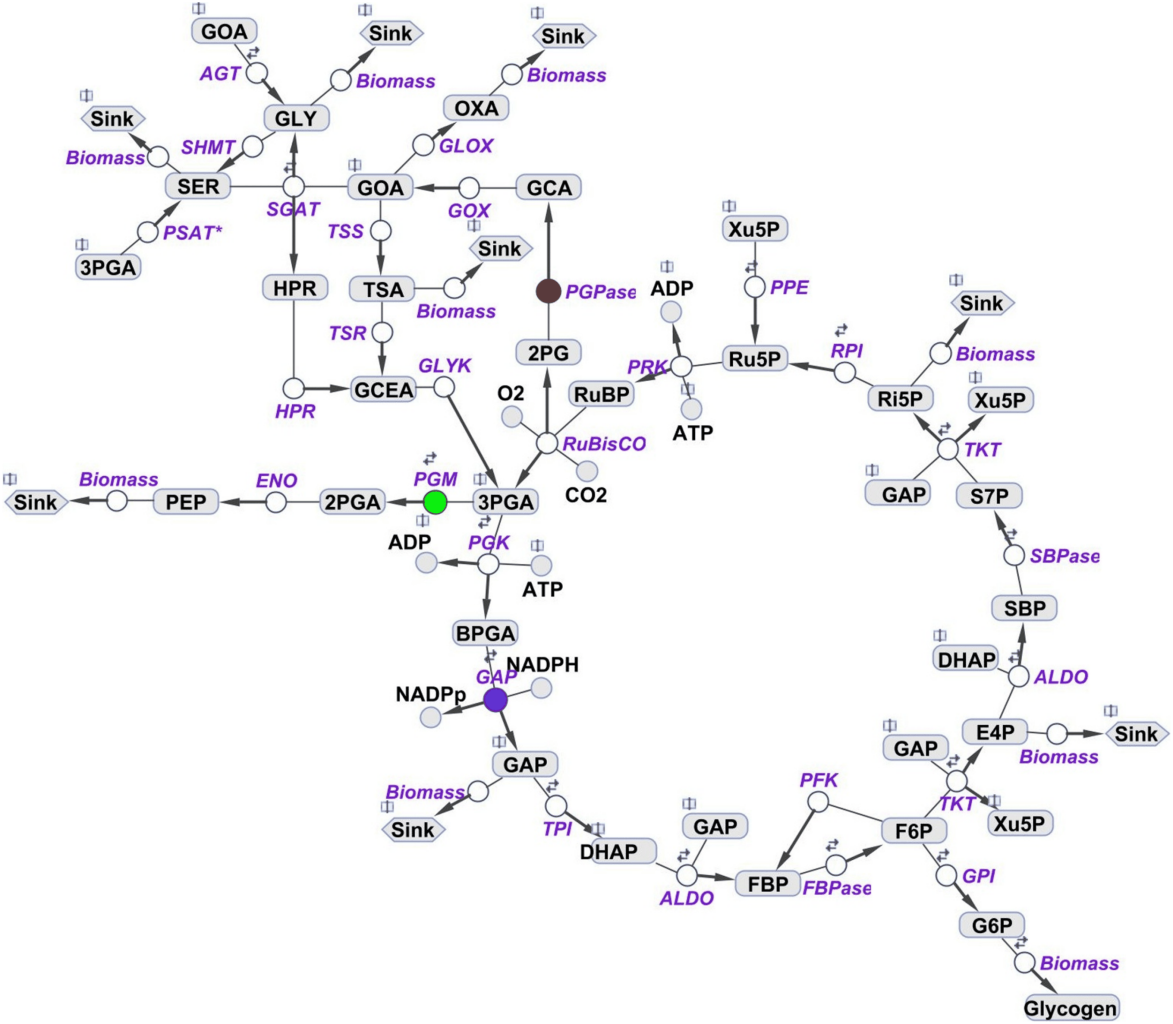
Scheme of primary carbon metabolism encoded as a kinetic model of *Synechococcus elongatus* PCC 7942. The model includes the Calvin-Benson cycle, glycogen synthesis, photorespiratory pathways, glycolysis, and sink reactions (representing the adjacent pathway and calculation of biomass production). Three reactions catalyzed by isozymes include phosphoglycerate mutases (green), glyceraldehyde-3-phosphate dehydrogenases (blue), and phosphoglycolate phosphatase (red). Reversibility of a particular reaction is indicated by two small arrows. For further details, see File S1. Purple color shows involved enzymes: RuBisCO-ribulose-1,5-bisphosphate carboxylase oxygenase, PGK-phosphoglycerate kinase, GAP-glyceraldehyde 3-phosphate dehydrogenase, TPI-triose phosphate isomerase, ALDO-aldolase, FBPase-fructose-1,6-bisphosphatase, PFK-phosphofructokinase, TKT-transketolase, SBPase-sedoheptulose-1,7-bisphosphatase, RPI-phosphopentose isomerase, PPE-phosphopentose epimerase, PRK-phosphoribulokinase, GPI-glucose-6-phosphat isomerase, PGPase-phosphoglycolate phosphatase, GOX-glycolate oxidase, SGAT-serine-glyoxylate transaminase, HPR-hydroxypyruvate reductase, GLYK-glycerate kinase, AGT-alanine-glyoxylate transaminase, TSS-tartronate semialdehyde synthase, TSA-tartronate semialdehyde reductase, SHMT-serine hydroxymethyltransferase, GLOX-glyoxylate oxidase, PSAT*-phosphoserine transaminase (3-phosphoglycerate dehydrogenase is, for simplicity, not implemented).

The presented multi-level kinetic model is based on Michaelis-Menten kinetics. The scheme of employed model is presented on Figure 1. A list of reactions, including V_max_ parameters and transcriptomic weight factors, is available in the file S1. The model versions for high and low CO_2_ are available in Model S1 and Model S2 in SBML L2V4 (XML). The model was developed, and simulations were executed using the SimBiology toolbox of MATLAB (Mathworks Inc.). The routine employed for parameter estimation was a hybrid genetic algorithm (ga_hybrid, Mathworks Inc.).

Relative transcriptomic and metabolomic data of *Synechococcus* 7942 cells grown at high CO_2_ (5% CO_2_) and low CO_2_ conditions (0.038% CO_2_) were taken from a previous study [11]. The consideration of two environmental conditions was necessary to understand and implement the changes in transcriptomic level of isozymes in the model and was essential for constraining the model by doubling the amount of metabolic data. To investigate the transitory phase of CO_2_ acclimation, we included a metabolic dataset for cells shifted from high to low CO_2_ conditions for 3 h (these unpublished data can be found in Table S1). These new data were obtained as described before [11].

**Figure 2:**
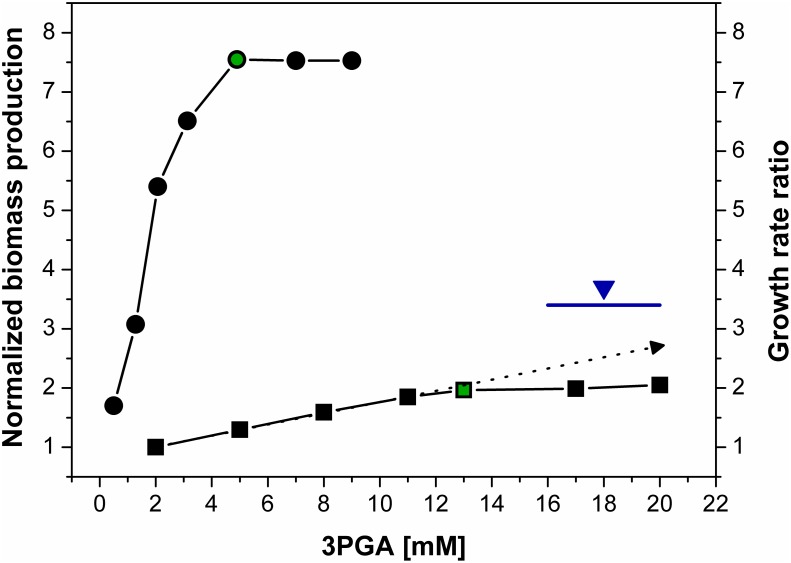
Simulated impact of 3PGA concentration on biomass production in *Synechococcus* 7942 cells grown under high and low CO_2_ conditions. **Black circles** indicate simulated data for high CO_2_, and the **green circle** indicates the experimental measurement. **Black squares** indicate simulated data for low CO_2_, and the **green square** indicates the experimental measurement. The **dotted arrow** indicates the slope between the linear and saturated regions. The **blue line** (measured) and **blue triangle** (simulated at the experimental level of 3PGA) represent the growth ratio of high and low CO_2_. The first circle corresponds to the first square, the second circle corresponds to the second square, etc., representing the coupled data describing a shift from high to low CO_2_.

The cellular concentration of 3-phosphoglycerate (3PGA) was recalculated compared with our previous study [10] using our own data. This change was a consequence of a self-validating process to improve multi-scale modelling, which predicted different levels of 3PGA. We found 3±1.6 mM 3PGA in the total cell volume. Assuming that approximately 60% of cyanobacterial cells represent the osmotic free accessible cytoplasm, 3PGA levels were calculated to be 5±2.7 mM in high CO_2_ grown cells. The developed model uses the mean value of 5 mM, which is validated by biomass efficiency estimation (Fig. 2). The concentrations of other metabolites were recalculated based on measured ratios relative to 3PGA [11] for the purpose of modeling.

## Results and Discussion

### Model description and validation

Presented multi-level kinetic model includes all enzymatic steps of the Calvin-Benson cycle, photorespiration, glycolysis, and simplified carbohydrate synthesis (Fig. 1). The model also includes sink reactions, e.g., Sink4 for tricarboxylic acid cycle (Fig. 1), representing the adjacent pathways and allows for the estimation of biomass production under changing CO_2_ conditions.

In general, the multi-scale kinetic model describes more than one layer of cellular function. To describe changing metabolic states under different environmental conditions within a model, information about changes in the amount of enzymes is necessary. Instead of proteomic data, which do not reflect all primary metabolism enzymes in a cyanobacterial cell [12], transcriptomic data were used to bridge the different steady states of enzyme abundances with corresponding metabolic levels. We assume that, in the case of prokaryotes, a change in gene expression in steady state is equal to change in enzyme activity which neglects possible impact of post-translation or other modifications. This assumption has been supported by a silencing experiment employed on 14 genes of another prokaryote, *Escherichia coli*. In this experiment [13], mRNA levels were reduced by at least 60% and, consequently, activities of corresponding enzymes decreased by at least 60%. In the case of reactions included in the presented model, genes for enolase, phosphoglycerate kinase and glucose-6-phosphat isomerase were silenced by 92%, 79% and 90% and respective enzyme activities decreased by 82%, 75% and 95% [13]. Taking in account the standard deviation of performed measurements [13], the possible differences between the change in gene expression and enzyme activity are between 0 and 16.3%.

Multi-scaling improves the accuracy of parameter fitting enormously because, compared with traditional kinetic modeling, it fits metabolic data for more than one steady state with the same kinetic parameters. In our case, we analyzed metabolic changes in cells of *Synechococcus* 7942 cultivated at high and low CO_2_ conditions. The model focuses on the major routes of primary carbon metabolism in *Synechococcus* 7942, as well as other phototrophic organisms that perform oxygenic photosynthesis.

The model has been validated for growth rate, metabolic, transcriptomics, redox and energy levels under two growing conditions: high and low CO_2_. The model validation on energy charge assumes the same ATP · (ADP+ATP)^−1^ ratio in high and low CO_2_, and was maintained in a narrow range 0.74–0.78 as proposed before [14]. The simulated redox level as NADPH · (NADPH+NADP^+^)^−1^ ratio in high and low CO_2_ corresponds with measured data (Burnap, personal communication) and reached level 0.33 for high CO_2_ and 0.43 for low CO_2_. The employed mean values for transcriptomic changes implies robustness of the whole system to parameter change and thus against the environmental changes. Finally, as shown in Table 1, the comparisons of the simulated and experimental values for the selected metabolites in cells under both high and low CO_2_ conditions correspond with each other.

**Table 1:** Comparison between the simulation and experiment metabolic concentrations in *Synechococcus* 7942 cells grown at high and low CO_2_.

high CO_2_	3PGA	2PGA	PEP	F6P	G6P	FBP	DHAP	2PG	GLY	SER	OXA	unit
experiment	4.99	0.13	0.21	2.51	3.63	0.02	0.01	0.37	0.83	1.06	1.80	mM
simulation	4.61	0.16	0.23	2.22	2.77	0.03	0.03	0.33	0.86	1.12	1.94	mM
**low CO_2_**	**3PGA**	**2PGA**	**PEP**	**F6P**	**G6P**	**FBP**	**DHAP**	**2PG**	**GLY**	**SER**	**OXA**	**unit**
experiment	13.00	0.62	0.98	3.00	4.05	0.07	0.01	0.33	1.11	1.34	1.29	mM
simulation	13.46	0.47	0.72	3.38	4.19	0.07	0.03	0.29	1.15	1.21	1.40	mM

3PGA–3-phosphoglycerate, 2PGA–2-phospohoglycerate, PEP–phosphoenolpyruvate, F6P–fructose 6-phosphate, G6P–glucose 6-phosphate, FBP–fructose 1,6-bisphosphate, DHAP–dihydroxyacetone phosphate, 2PG–2-phosphoglycolate, GLY–glycine, SER–serine, OXA–oxalate. All values were rounded to the nearest second decimal.

The employed systems biology workflow for identification of kinetic parameters has several steps. The first step is a search for various sets of kinetic parameters matching the metabolic data from cells grown at high CO_2_. Next step includes a switch from high to low CO_2_ and applying the mean values of measured transcript changes as weight factors for each V_max_ estimated for high CO_2_. The third step simulates a transition to steady state in low CO_2_ and evaluates the match with experimental data from cells grown at low CO_2_. If no match is found after hundreds of parameter estimation runs, the model is re-evaluated and missing reaction(s) or regulatory steps, e.g., izoenzyme, are implemented. Finally, if the match is found, it is the first step for the model validation, followed by comparing calculated and measured energy and redox levels and growth rate ratio between high and low CO_2_ states.

### Prediction of cellular metabolic concentration at different CO_2_ conditions

Different levels of CO_2_ greatly impact metabolic concentrations [11], [15] and gene expression [16], [17], [18]. However, it is difficult to explain what regulates specific metabolic concentrations in cells exposed to a changing environment without a systems biology approach. To answer this question, we first asked whether it was possible to predict and explain the absolute metabolic concentration of 3-phosphoglycerate (3PGA), the key metabolite in primary carbon metabolism (Fig. 1). The flux of 3PGA between the Calvin-Benson cycle and glycolysis is regulated by isozymes phosphoglycerate mutases (PGMs). Since we have shown that one-isoenzyme scenario cannot keep the balance between 3PGA and 2PGA [10], the prerequisite for such analysis is the implementation of PGM 1, 2, and 3 into the model.

We focused on a broad range of 3PGA concentrations around the measured levels (see Materials and methods). We calculated concentrations of other metabolites based on measured relative ratios [11] for each particular concentration of 3PGA within the chosen range. Then, we applied redox (NADPH) and energetic (ATP) constraints and ran the parameter estimation to fit the metabolic dataset in high CO_2_ and checked whether the following simulation in low CO_2_ matched the changes in metabolome as well. In the case of positive result, we stored the result and repeated it many times in order to get the highest biomass production. The final step was a search for any trend allowing to predict the absolute concentration of 3PGA. We note that the biomass production in the model means the accumulation of carbon atoms in sinks, i.e., pyruvate has 3 carbons so it contribute less than ribulose with 5 atoms; the nitrogen metabolism is not included in the model but it is not a limiting under our growth conditions [11].

Comparing all acquired data from high and low CO_2_ revealed a clear trend. Increasing cellular 3PGA concentrations have a positive impact on biomass production at both CO_2_ conditions and reveal a clear trend and a connection between 3PGA accumulation and biomass production, as shown on Figure 2. Furthermore, the reduction of 3PGA below the threshold level of 2.5 mM resulted in dramatically decreased biomass production (Fig. 2). This interdependence explains why 3PGA is tightly regulated to maintain specific concentrations under varying CO_2_ conditions [10]. Lower 3PGA levels slow down the growth and higher levels have no (high CO_2_) or a negligible impact on growth rate (low CO_2_). Taken together, the model predicts the absolute concentration of 3PGA at the level providing the highest biomass production. This conclusion is supported by experimentally estimated concentrations of 3PGA, which are positioned on both calculated curves at the very beginning of the saturated region (Fig. 2).

### Role of glyceraldehyde-3-phosphate dehydrogenases in metabolic and redox regulation

In addition to PGM, there is another isozyme in primary carbon metabolism, glyceraldehyde-3-phosphate dehydrogenase (GAP). GAP catalyzes the conversion between glyceraldehyde 3-phosphate (G3P) and glycerate 1, 3-bisphosphate (Fig. 1). Compared with PGM, which has a cardinal position [10] in the metabolic crossroad between the Calvin-Benson cycle and glycolysis in cyanobacteria (Fig. 1), GAP is localized in the linear part of the reaction chain (Fig. 1). Thus, we investigated the role of GAP in this “non-prominent” position.

In most cyanobacteria, three genes encoding for GAP isozymes (*gap1*, *gap2*, and *gap3*) have been annotated. NAD-dependent GAP1 (synpcc7942_0245) was reported to primarily act as a glycolytic enzyme, whereas GAP2 operates within the Calvin-Benson cycle [19]. The role of GAP3 (gene synpcc7942_1742) has not yet been identified [20]. Due to sequence similarity, we have concluded that *gap1* and *gap3* encode isozymes responsible for glycolytic glucose conversion. Our model analysis of glycolytic GAPs showed that a simulated double knock-out of GAP1 and GAP3 significantly impacts biomass production, leading to a 24.3% decrease of biomass in ambient air but only a 4% decrease in the high CO_2_ condition. Interestingly, the simulated knock-out of either GAP1 or GAP3 had negligible (approximately 2%) impact on biomass production in ambient air. This finding corresponds well with the fact that GAP3 is missing in some cyanobacteria (e.g., *Synechocystis* sp. PCC 6803). Thus, we focused on the role of the *Synechococcus* 7942 GAP3.

**Figure 3:**
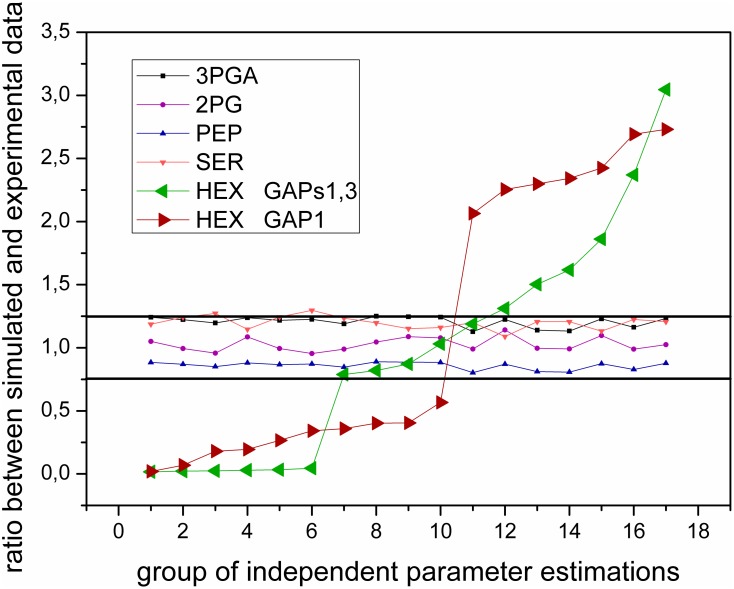
Impact of single GAP1 and dual GAP 1,3 regulation on metabolic levels cells of *Synechococcus* 7942. Match between simulated and measured data in low CO_2_, depending on the estimated kinetic parameters (V_max_, k_m,_ and k_eq_values) in high CO_2_ for GAP1 (**red color**) and GAPs1,3 (**green color**). HEX represents the mean value of sum of F6P, FBP, and G6P; their values were nearly identical, and this simplification was made to minimize the number of curves in one figure without losing any information. The black lines indicate ±25% difference region around the hypothetical perfect match value. Note: Parameter estimations are independent (similar to repeating experiments). However, to see the trend, the data were arranged from the lowest to the highest value. Every point represents many repetitions in parameter estimation that give the same result, which should identify all combinations and thus implies the existence of bifurcation behavior.

Our pilot analysis showed that GAP3 also has a negligible impact on its substrate and product. The common approach for identifying the function of any enzyme is a sensitivity analysis to reveal which metabolites are sensitive to changes of a particular enzymatic activity [21]. However, this method is not applicable for isozymes in prokaryotic cells because the system is not sensitive to small changes in GAP3 activity due to the influence of GAP1 and vice-versa. To determine which part of metabolism is sensitive to GAP3 activity changes, we varied the parameter space of the model to match data in the high CO_2_ condition and analyzed the impact of different parameter sets on the system behavior in the low CO_2_ condition. We have previously shown that this method is able to reveal trends in iso-enzymatic regulation [10]. In comparison to our previous work with PGMs, a trend was not directly visible after plotting the results. We note that this is not a problem of employed method but a consequence of different roles of particular isozymes in different location in metabolism. In order to see the trend in this case, it was necessary to arrange the data from the lowest to the highest value (Fig. 3).

This analysis identified one group of metabolites, the hexoses, that was sensitive to GAP changes, whereas most of the metabolites tested were insensitive to GAP changes (Fig. 3). For simplicity, concentrations of fructose 6-phosphate (F6P), glucose 6-phosphate (G6P), and fructose 1, 6-bisphosphate (FBP) were summed as hexoses, and individual curves of F6P, G6P, and FBP yielded practically identical trends. The bifurcation point (sudden qualitative change in behavior) can be clearly observed in both scenarios. However, the addition of GAP3, i.e., cooperation of two glycolytic GAPs, shifts its position and allows a perfect match with the experimental data (Fig. 3). Since no regulatory impact on other metabolites was observed, this result suggests a specific role of GAP3 in metabolic regulation of hexose levels to improve the control over carbohydrate synthesis, especially under changing environmental conditions. Finally, this result also implies that our “trend search method” is applicable to isozymes other than PGMs.

**Figure 4:**
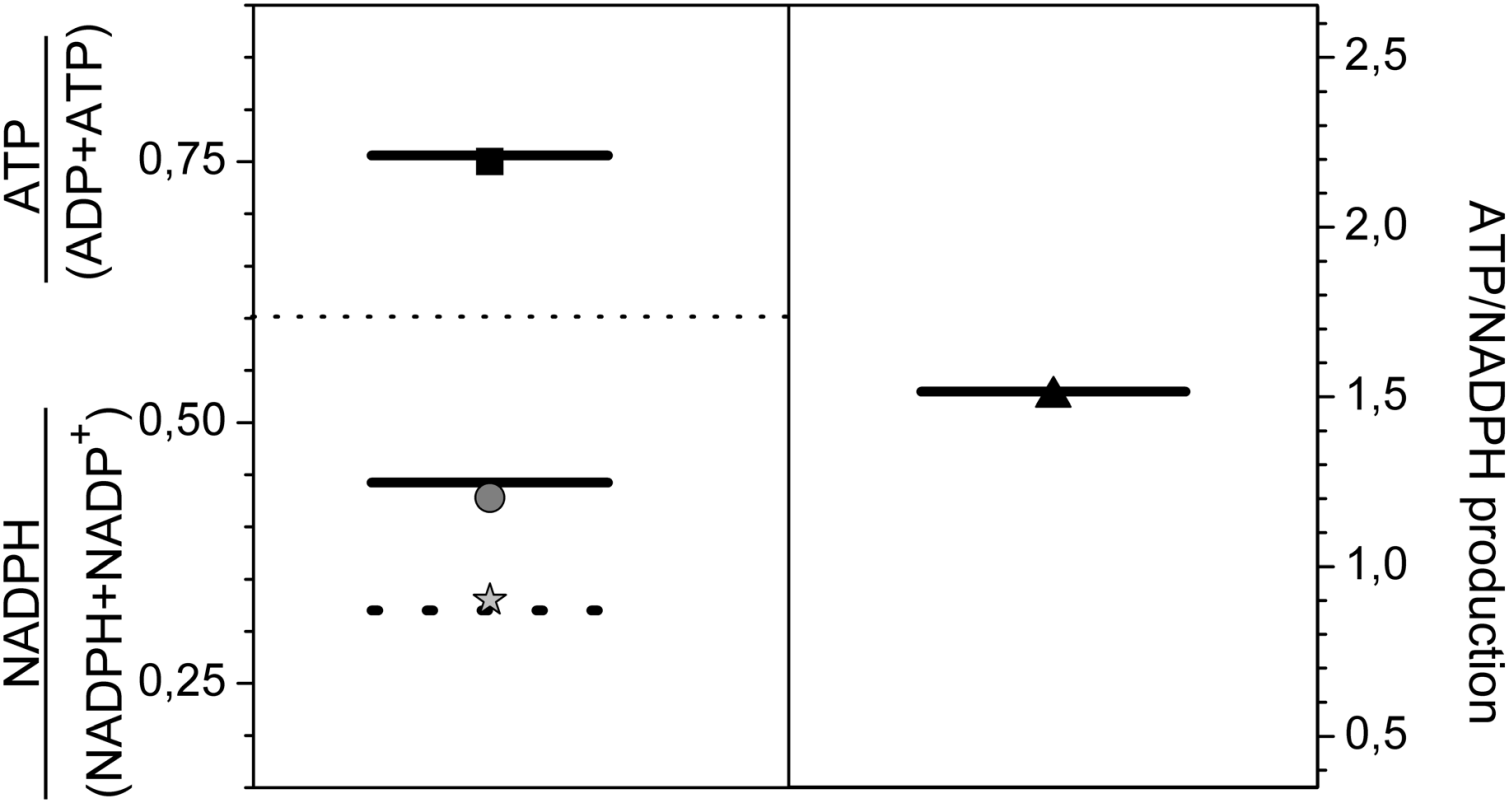
Homeostasis of ATP and NADPH in *Synechococcus* 7942 cells under changing CO_2_ conditions. The upper left corner shows the match between the simulated (**black line**) and experimental values (**solid square**) of the ATP · (ADP+ATP)^−1^ ratio at both high and low CO_2_. The lower left section indicates the NADPH · (NADPH+NADP^+^)^−1^ ratio: **grey circle** for low CO_2_; **grey asterisk** for high CO_2_, **grey dotted line** for simulation in low CO_2_, with neglecting NAD co-regulation in the reaction catalyzed by GAPs, **grey line** for simulation in low CO_2_ with NAD replaced by NADPH in a reaction catalyzed by GAPs. The right section shows the measured and simulated ratio of ATP/NADPH production.

Moreover, we also found indications that the isozymes GAP1 and GAP3 influence the redox level in *Synechococcus* 7942. The NADPH/NADP^+^ ratio was 50% in cyanobacterial cells at high CO_2_ and increased to 75% at low CO_2_ conditions (Robert Burnap, Oklahoma State University, USA, personal communication). We tested whether the lower flux of NADPH due to cyclic electron transport around photosystem I at low CO_2_ [22], [23] could explain this difference, but the result was negative. We note that decreased NADPH production due to cyclic electron transport around photosystem I at low CO_2_ remains implemented in the model. Furthermore, the test scenario revealed that neglecting the possible NADH regulation of GAP1 and 3 does not allow for the aforementioned measured changes in the NADPH/NADP^+^ ratio. However, it is possible that the two glycolytic GAPs do not exclusively use NADH but could be NADPH-dependent in the non-compartmented cyanobacterial cell [24]. Assuming NADPH-dependence for these two GAPs not only perfectly matched the changing NADPH/NADP^+^ ratios under both CO_2_ conditions (Fig. 4, black arrow) but also reflected the 3∶2 ratio for ATP/NADPH production known for photosynthetic organisms [25], [26]. Taken together, the model predicts that the 25% increase of reduced NADPH under ambient air is primarily caused by glycolytic GAPs as a result of 3PGA accumulation in cells at ambient air conditions (Fig. 2).

### How is photorespiration integrated into primary carbon metabolism?

Photorespiratory metabolism converts the toxic oxygenase reaction product of Rubisco, 2-phosphoglycolate (2PG), back into the Calvin-Benson cycle intermediate 3PGA. Despite the occurrence of an active CO_2_ concentrating mechanism, photorespiration is essential for cyanobacteria under ambient air conditions [15]. However, the reason for the essential nature of photorespiration among cyanobacteria is difficult to understand because its overall rate is low [7], [27], and glycolate is believed to be excreted from cyanobacterial cells [28]. The inclusion of photorespiration into our model of primary carbon metabolism allowed the analysis of its role in and implementation into overall metabolism *in silico*.

**Figure 5:**
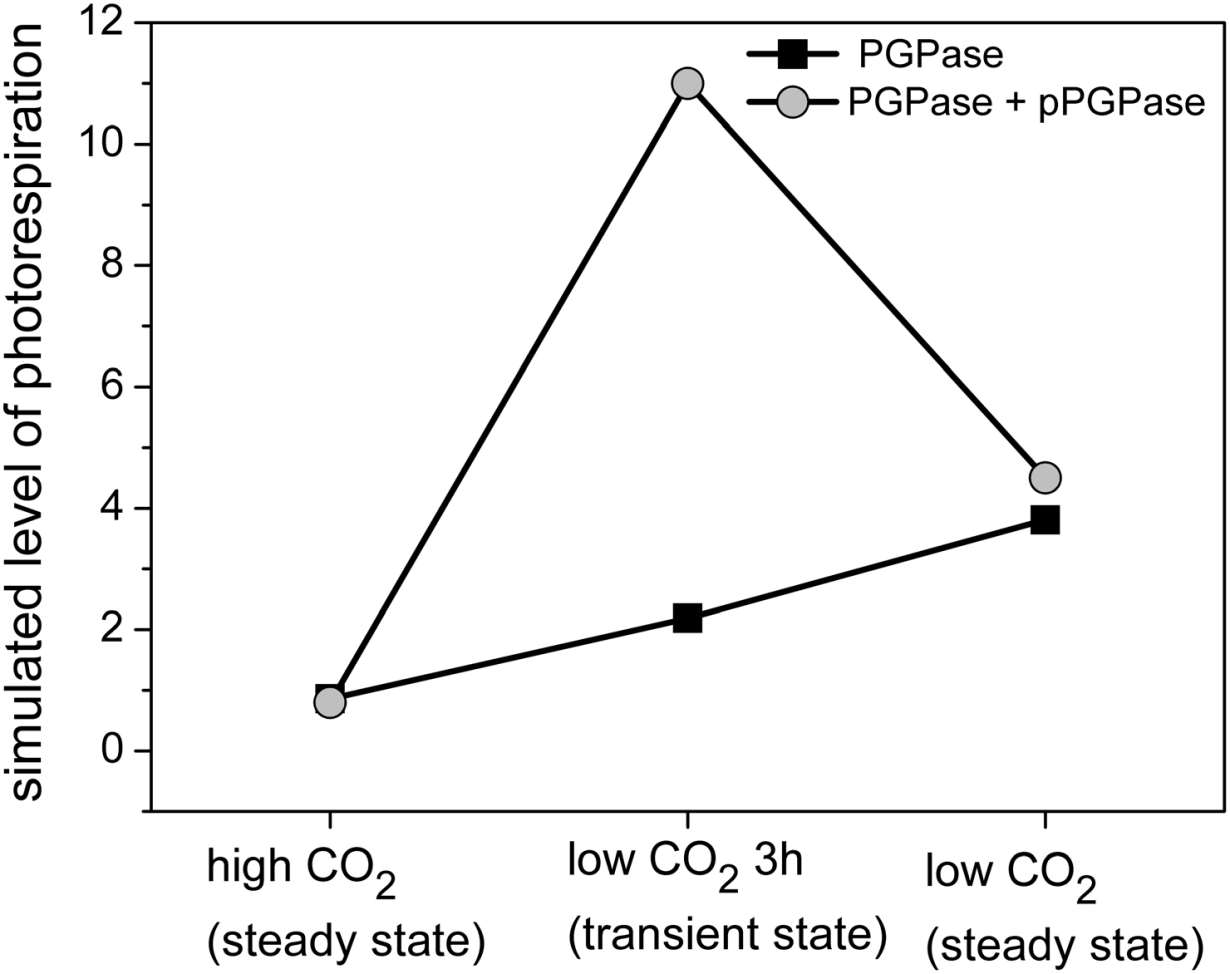
Photorespiratory level in *Synechococcus* 7942 cells at changing CO_2_ levels. PGPase–2-phosphoglycolate phosphatase, pPGPase–putative PGPase. Photorespiratory level– the percentage of RuBisCO capacity used by its oxygenase activity. Figure represents two scenarios of the same high → low CO_2_ transition. Two steady states and one transient state are shown.

First, the role of isozymes of 2PG phosphatase (PGPase) was analyzed. In contrast to PGM and GAP isozymes, which perform an important role in steady state metabolic regulation, the estimated level of photorespiration in cells grown at high or low CO_2_ steady states is not sensitive to the presence of putative PGPase (Fig. 5). It was unclear why two PGPases are needed to perform the photorespiration. Uncertainty regarding annotated putative PGPases (annotated PGPase synPCC7942_2613, putative PGPase synPCC7942_0217) makes this question even more interesting. Regarding the role and number of PGPases, it should be noted that the substrate 2PG of PGPase inhibits certain enzymes of the Calvin-Benson cycle [29]. Hence, it is essential to reduce the concentration of 2PG.

Thus far, only steady states at high and low CO_2_ have been considered. Thus, we analyzed additional transcriptomic [11] and metabolic (Table S1) data from the transition phase (3 h after shifts from high to low CO_2_). Assuming the activity of only one PGPase was able to match the level of 2PG but resulted in a gradual increase of photorespiration (Fig. 5, black). However, matching the level of 2PG with the combined action of two PGPases induced a transient spike in the photorespiratory level (Fig. 5, gray). In other words, one PGPase predicts a gradual increase of photorespiration, while two PGPases suggests a significant increase in Rubisco oxygenase activity after shifting to low CO_2_ conditions. Since it is has been shown that 1) the photorespiratory flux particularly increases during the transition phase of shifts from high to low CO_2_ [27] and 2) cyanobacteria stop growing during the transition phase [11], it can be concluded that one PGPase is not able to efficiently remove the toxic 2PG in the transient state, i.e., under changing conditions. Thus, our analysis of iso-enzymatic regulation validates the annotation for more than one putative PGPase and explains the need for two PGPases.

Finally, our analysis supported the notion that cyanobacteria, similar to other oxygenic phototrophs, perform better with lower or even no photorespiration. However, it is unclear why all cyanobacteria harbor the genomic capacity for photorespiration and do not release the low levels of photorespiratory glycolate from the cell. If we take our estimation of photorespiration [27] to be approximately 4.5% of the overall capacity of RuBisCO, we can directly calculate the costs of cutting photorespiration out of metabolism. The decrease in biomass production at low CO_2_ conditions (ambient air) was 11.3% without active photorespiratory pathway. This lower productivity is partially due to losing organic carbon in the form of glycolate and partially by rebalancing metabolism, namely the phosphoserine pathway. However, the 11.3% calculated biomass decrease is valid only for ATP non-limited systems and can increase to 18% under ATP-(light)-limiting conditions in the natural environment. This growth reduction may partially explain why all cyanobacteria kept the photorespiratory pathway in addition to its role of detoxifying critical intermediates, such as 2PG.

## Conclusions

The main goal of this work is to show the diversity of iso-enzymatic regulatory roles in prokaryotes and to show how to decipher the functions of particular isozyme. The essential part for such analysis is combining metabolic and transcriptomic data within the multi-level kinetic model. In our study we have focused on *Synechococcus* 7942 cultivated at high and low CO_2_ conditions and two isozymes from central carbon metabolism, glyceraldehyde-3-phosphate dehydrogenase and 2PG phosphatase. The heterogeneity of their roles includes boosting the glycolytic flux, tuning the hexose regulation and protection against toxic substrate in changing environment. Finally, we also showed that complex model is able to predict the absolute concentration of metabolites, in our case shown for the first stable molecule of carbon synthesis, 3-phosphoglycerate.

The presented multi-level kinetic model introduces a new methodology identifying the iso-enzymatic regulation which will be employed for main cyanobacterial model organism, *Synechocystis* sp. PCC 6803, and eventually for other prokaryotes. Starting with *Synechococcus* 7942 reduces a risk of errors for more “prominent” species, cross-validate the gene annotation between species, double amount of processed metabolic and transcriptomic data and enable to explain the difference between both species, and eventually among other cyanobacteria and bacteria.

Finally, it might be possible to partially validate the model-based prediction by knocking down certain isozyme and making a metabolic and transcriptomic screening for at least two different conditions. Moreover, the model predicts changes in the level of metabolites, which are currently not quantified by the GC-MS-based metabolome protocol. Efforts to quantify those metabolites by alternative methods would represent another strategy for future model validation.

## Supporting Information

Table S1 Metabolic data for transition phase 3 h after shifts from high to low CO_2_.
(XLS)

File S1 A list of reactions, including V_max_ parameters and transcriptomic weight factors.
(DOC)

Model S1 The model of primary carbon metabolism in cyanobacterium Synechococcus elongatus PCC 7942 for high CO_2_ condition.
(XML)

Model S2 The model of primary carbon metabolism in cyanobacterium Synechococcus elongatus PCC 7942 for low CO_2_ condition.
(XML)
