# Supplementary material for: Multi-Level Kinetic Model Explaining Diverse Roles of Isozymes in Prokaryotes
Source: PLoS One. 2014 Aug 15;9(8):e105292. doi: 10.1371/journal.pone.0105292 (PMC4138046; doi:10.1371/journal.pone.0105292)
Supplement: File S1 — A list of reactions, including Vmax parameters and transcriptomic weight factors. (DOC) [file pone.0105292.s002.doc]

**Supplement S1: Model description**

Table 2 **The list of reactions and major kinetic parameters included in multi-kinetic model of carbon prime metabolism for *Synechococcus* 7942.** Vm HC indicates the Vmax, estimated for high CO_2_ condition; this does not apply for the first three reactions which are described by mass action law and thus Vm HC has a meaning of rate constant. WF LC indicates the weight factor multiplying Vm for low CO_2_, taken^12^ as mean value of transcriptomic changes between high and low CO_2_. RuBP – ribulose 1,5-bisphospate, 3PGA – 3-phosphoglycerate, BPGA – 1,3-bisphosphoglycerate, GAP – glyceraldehyde 3-phosphate, DHAP – dihydroxyacetone phosphate, FBP – fructose 1,6-bisphosphate, F6P – fructose 6-phosphate, E4P – erythrose 4-phosphate, Xu5P – xylulose 5-phosphate, SBP – sedoheptulose 1,7-bisphosphate, S7P – – sedoheptulose 7-phosphate, Ri5P – ribose 5-phosphate, Ru5P – ribulose 5-phosphate, 2PGA – 2-phospohoglycerate, PEP – phosphoenolpyruvate, 2PG – 2-phosphoglycolate, GCA – glycolate, GOA – glyoxylate, SER – serine, HPR – hydroxypyruvate, GLY – glycine, GCEA – glycerate, OXA – oxalate, TSA – tartronate-semialdehyde, G6P – glucose 6-phosphate, G1P – glucose 1-phosphate, THR – threonine.

| **model ID** | **reaction** | **Vm HC** | **WF LC** | **pathway** |
| --- | --- | --- | --- | --- |
| Light_1 | ADP + Pi → ATP | 0.19 | 0.13 | light reactions |
| Light_2 | NADPp → NADPH | 0.68 | 0.21 | light reactions |
| CO2 dif | extCO2 → cytCO2 | 1.91 | 0.093 | CO2 transport |
| CC_1 | RuBP + CO2 → 2*3PGA | 0.991 | 0.963 | Calvin-Benson cycle |
| CC_2 | 3PGA + ATP → BPGA + ADP | 0.6 | 0.35 | Calvin-Benson cycle |
| CC_3 | BPGA + NADPH ↔ GAP + NADPp + Pi | 0.53 | 0.07 | Calvin-Benson cycle |
| CC_4 | GAP ↔ DHAP | 0.1 | 0.13 | Calvin-Benson cycle |
| CC_5 | GAP + DHAP ↔ FBP | 1.61 | 0.1 | Calvin-Benson cycle |
| CC_6 | FBP → F6P + Pi | 1.23 | 1.67 | Calvin-Benson cycle |
| CC_7 | F6P + GAP ↔ E4P + Xu5P | 0.3 | 0.33 | Calvin-Benson cycle |
| CC_8 | DHAP + E4P ↔ SBP | 1.11 | 0.1 | Calvin-Benson cycle |
| CC_9 | SBP → S7P + Pi | 0.16 | 0.87 | Calvin-Benson cycle |
| CC_10 | S7P + GAP ↔ Ri5P + Xu5P | 0.23 | 1.67 | Calvin-Benson cycle |
| CC_11 | Ri5P ↔ Ru5P | 1.3 | 1.07 | Calvin-Benson cycle |
| CC_12 | Xu5P ↔ Ru5P | 1.68 | 0.14 | Calvin-Benson cycle |
| CC_13 | Ru5P + ATP → RuBP + ADP | 0.43 | 0.33 | Calvin-Benson cycle |
| GL_1 | F6P → FBP | 0.24 | 2.71 | Glycolysis |
| GL_2a | GAP + NADPp + Pi ↔ BPGA + NADPH | 0.82 | 3.05 | Glycolysis |
| GL_2b | GAP + NADPp + Pi ↔ BPGA + NADPH | 2.44 | 0.94 | Glycolysis |
| GL_3a | 3PGA ↔ 2PGA | 0.64 | 15.42 | Glycolysis |
| GL_3b | 3PGA ↔ 2PGA | 1.9 | 0.6 | Glycolysis |
| GL_3c | 3PGA ↔ 2PGA | 1.59 | 0.71 | Glycolysis |
| GL_4 | 2PGA ↔ PEP | 1.69 | 0.43 | Glycolysis |
| PP_1 | RuBP + O2 → 2PG + 3PGA | 0.009 | 5 | Photorespiration |
| PP_2a | 2PG -> GCA | 0.001 | 2 | Photorespiration |
| PP_2b | 2PG -> GCA | 0.002 | 0.73 | Photorespiration |
| PP_3 | GCA -> GOA | 0.0042 | 2 | Photorespiration |
| PP_4 | GOA + SER <-> HPR + GLY | 0.43 | 0.42 | Photorespiration |
| PP_5 | 2*GLY -> SER | 0.002 | 0.87 | Photorespiration |
| PP_6 | HPR -> GCEA | 0.004 | 0.4 | Photorespiration |
| PP_7 | GCEA + ATP -> 3PGA + ADP | 0.05 | NA | Photorespiration |
| OX_1 | GOA -> OXA | 0.25 | NA | oxalate pathway |
| GC_1 | GOA -> TSA | 0.01 | NA | glycerate pathway |
| GC_2 | TSA -> GCEA | 0.1 | NA | glycerate pathway |
| GSM_1 | GOA -> GLY | 0.1 | 0.81 | GLY, SER metabolism |
| GSM_2 | 3PGA -> SER | 0.0018 | NA | GLY, SER metabolism |
| GSM_3 | GLY -> THR | 0.0005 | 0.67 | GLY, SER metabolism |
| SS_1 | F6P <-> G6P | 0.05 | 0.66 | carbohydrates synthesis |
| SS_2 | G6P <-> Glycogen | 0.0009 | 0.00017 | carbohydrates synthesis |
| EXP_1 | GCA -> out | 0 |  | tested channel out of cell |
| Sink_X | DHAP, E4P, Ri5P, PEP ... -> Sink | 0 - 0.85 |  | adjacent pathways for biomass |
